# Triorganotin Derivatives Induce Cell Death Effects on L1210 Leukemia Cells at Submicromolar Concentrations Independently of P-glycoprotein Expression

**DOI:** 10.3390/molecules23051053

**Published:** 2018-05-01

**Authors:** Viera Bohacova, Mario Seres, Lucia Pavlikova, Szilvia Kontar, Martin Cagala, Pavel Bobal, Jan Otevrel, Julius Brtko, Zdena Sulova, Albert Breier

**Affiliations:** 1 Institute of Molecular Physiology and Genetics, Centre of Bioscience, Slovak Academy of Sciences, Dubravska Cesta 9, 84005 Bratislava, Slovakia; viera.bohacova@savba.sk (V.B.); mario.seres@savba.sk (M.S.); lucia.pavlikova@savba.sk (L.P.); szilvia.kontar@savba.sk (S.K.); martin.cagala@savba.sk (M.C.); 2 Department of Chemical Drugs, Faculty of Pharmacy, University of Veterinary and Pharmaceutical Sciences, Brno, Palackeho 1946/1, 61242 Brno, Czech Republic; bobalp@vfu.cz (P.B.); otevrelj@vfu.cz (J.O.); 3 Institute of Experimental Endocrinology, Biomedical Research Center SAS, Dubravska Cesta 9, 84505 Bratislava, Slovakia; julius.brtko@savba.sk; 4 Institute of Biochemistry and Microbiology, Faculty of Chemical and Food Technology, Slovak University of Technology, Radlinskeho 9, 81237 Bratislava, Slovakia

**Keywords:** L1210 cells, P-glycoprotein, multidrug resistance, triorganotin derivatives, apoptosis, calcein cell retention

## Abstract

The acceleration of drug efflux activity realized by plasma membrane transporters in neoplastic cells, particularly by P-glycoprotein (P-gp, ABCB1 member of the ABC transporter family), represents a frequently observed molecular cause of multidrug resistance (MDR). This multiple resistance represents a real obstacle in the effective chemotherapy of neoplastic diseases. Therefore, identifying cytotoxic substances that are also effective in P-gp overexpressing cells may be useful for the rational design of substances for the treatment of malignancies with developed MDR. Here, we showed that triorganotin derivatives—tributyltin-chloride (TBT-Cl), tributyltin-bromide (TBT-Br), tributyltin-iodide (TBT-I) and tributyltin-isothiocyanate (TBT-NCS) or triphenyltin-chloride (TPT-Cl) and triphenyltin-isothiocyanate (TPT-NCS)—could induce the death of L1210 mice leukemia cells at a submicromolar concentration independently of P-gp overexpression. The median lethal concentration obtained for triorganotin derivatives did not exceed 0.5 µM in the induction of cell death of either P-gp negative or P-gp positive L1210 cells. Apoptosis related to regulatory pathway of Bcl-2 family proteins seems to be the predominant mode of cell death in either P-gp negative or P-gp positive L1210 cells. TBT-Cl and TBT-Br were more efficient with L1210 cells overexpressing P-gp than with their counterpart P-gp negative cells. In contrast, TBT-I and TPT-NCS induced a more pronounced cell death effect on P-gp negative cells than on P-gp positive cells. Triorganotin derivatives did not affect P-gp efflux in native cells measured by calcein retention within the cells. Taken together, we assumed that triorganotin derivatives represent substances suitable for suppressing the viability of P-gp positive malignant cells.

## 1. Introduction

Important progress in the development of modern protocols for the targeted therapy of cancer was achieved during the past decade [1]. Additionally, the targeting of neoplastic cells with small molecules that could induce the elimination of neoplastic cells with a specific phenotype represents an important issue for biomedical research [2]. Nevertheless, even if the impressive responses of these therapies were fulfilled, several individuals are non-responders and show signs of drug resistance that may occur due to the phenotypic plasticity of cancer cells [3]. Neoplastic cells use several mechanisms to escape from the cell death effects of anticancer drugs [4]. Malignant cells could develop resistance to a wide group of structurally unrelated substances with different mechanisms of anticancer action—multidrug resistance (MDR) [5]. The overexpression of P-glycoprotein (P-gp), a drug efflux pump of the plasma membrane (ABCB1 member of ABC transporter gene family), represents the most frequently observed molecular cause of MDR [6]. A typical feature of P-gp represents broad substrate specificity to large but well-defined groups of structurally unrelated chemicals belonging to a cluster of P-gp substrates. When expressed in neoplastic cells, P-gp could cause massive drug resistance to its substrates involving anthracyclines (e.g., doxorubicin), Vinca alkaloids (e.g., vincristine VCR), actinomycines (e.g., actinomycin D, dactinomycines), taxols (e.g., paclitaxel), alkylating agents (mitomycin C), peptide antibiotics (gramicidin, valinomycin), and many others [7]. Several substances, including P-gp substrates, are known as P-gp inducers, i.e., substances that elevate P-gp expression. Thus, identifying substances that suppress malignant cell growth and are not P-gp-substrates and/or P-gp-inducers represents an important assignment for medicinal chemistry research.

The anticancer activity of triorganotin derivatives (tributyltin—TBT and triphenyltin—TPT) at submicromolar concentrations was recently shown on human breast cancer cell lines MCF-7 and MDA-MB-231 [8]. This effect may be linked to the depression of cell growth due to induction of p53 and Bcl-2 protein family related apoptosis [9]. Due to the induction of progesterone biosynthesis, effects on aromatase activity, and the capability of inducing the transcriptional activity of thyroid hormone receptor, these compounds are endocrine-disrupting agents [10,11,12]. Moreover, as retinoid X receptor ligands, these compounds may induce multimodal alterations of the cell phenotype [13].

Triorganotins represent organometallic small molecules that have been introduced to the environment as pesticides and antifouling agents in boat paint [14]. At a higher pH, organotins form stable uncharged complexes with -Cl, -Br, -I and -OH (R_3_SnOH^0^ or R_3_SnCl(Br, I)^0^), which also exhibit extensive hydrophobic (lipophilic) properties [15]. At a lower pH, organotins form cations (R_3_Sn+) with pK_a_ values of 6.25 and 5.2 for TBT and TPT, respectively [16]. Triorganotin chlorides are stable substances in aqueous mediums at close neutral pH. This may be documented by t_1/2_ = 4950 days (13.6 years) and t_1/2_ = 11,550 days (31.6 years) for TPT-Cl and TBT-Cl obtained from degradation kinetic parameters [17]. Even if bromo-, iodo- and isothiocyanato- derivatives may be less stable, rapid decomposition in aqueous mediums at close neutral pH could not be expected.

Relatively little is known about the effects of triorganotin derivatives on P-gp expression/efflux activity in neoplastic cells. Tributyltin, administered via the placenta and lactation, significantly reduced the expression of the renal *abcb1b* gene (*mdr1b*, gene encoding ABCB1b isoform of P-gp) in offspring [18]. However, after prolonged treatment, TBT increased P-gp expression/efflux activity in a monolayer of human intestinal Caco-2 cells [19].

In the present study, we examined the effects of TBT derivatives (chloride, bromide, iodide and isothiocyanate) and TPT derivatives (chloride and isothiocyanate) on mouse leukemia cells that were not expressing and expressing P-gp. As a cell model, we used parental P-gp negative mouse lymphocytic leukemia cells L1210 (S) and their two P-gp positive variants: R obtained by selection of S cells with VCR [20] or T obtained by transfection of S cells with the human gene encoding P-gp [21].

## 2. Results

### 2.1. Characterization of P-gp Positive Variants of L1210 Cells

P-gp positive variants of L1210 cells (R and T) strongly express P-gp either at mRNA or protein levels compared with previously described S cells [21]. Moreover, in this same study, we also demonstrated P-gp efflux activities in R and T cells and a lack of this activity in S cells using calcein retention assay. Both R and T cells are strongly less sensitive to P-gp substrates, such as VCR, doxorubicin and mitoxantrone, compared with S cells [22]. These features were periodically controlled for S, R and T cells during the experimental period in the present study. Taken together, S, R and T cells could be considered as suitable cell models for studying the differences between the responses of P-gp negative and P-gp positive leukemia cells to various chemicals.

### 2.2. Cytotoxic Effect of TBT and TPT Derivatives on S, R, T and PB-1 Cells

In the present study, we used the six triorganotin derivatives shown in Table 1. The lipophilicity of these substances is expressed as the log P (logarithm of partition coefficient in n-octanol-water.

**Figure 1:**
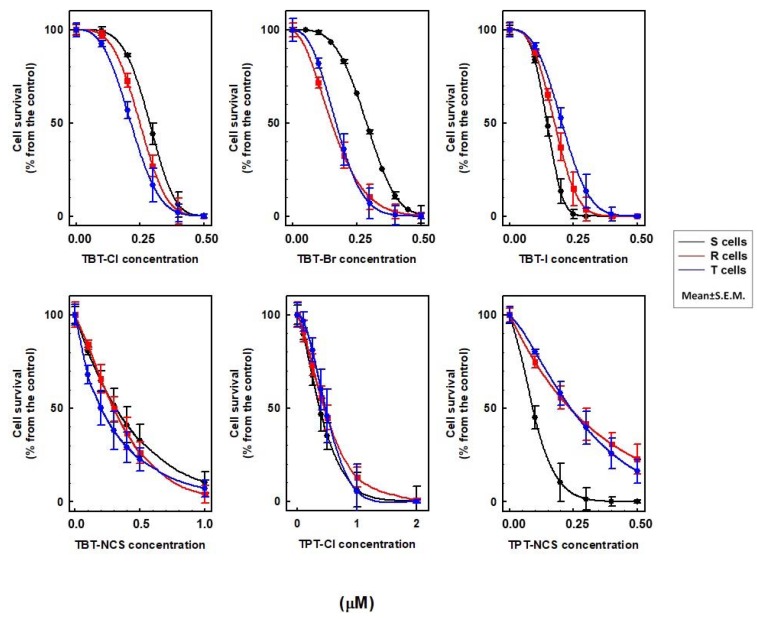
The cell death effects of triorganotin derivatives on S, R and T cells. The cells’ prior measurements were 48 h cultivated in cultivation medium in the absence or presence of the respective triorganotin derivatives at different concentrations. The data were fitted by nonlinear regression according to Equation (1) using SigmaPlot 8.0 software (Systat Software, Inc., San Jose, CA, USA). The data represent the means ± S.E.M. from six independent measurements corresponding IC_50_ values are summarized in Table 2.

At submicromolar concentrations, all triorganotin derivatives induced cell death effects on S, R and T cells (Figure 1). TBT-Cl and TBT-Br show higher effects on P-gp positive R and T cells than on P-gp negative S cells. In contrast, TBT-I and TPT-NCS more effectively depressed the cell viability of S cells than that of R and T cells. TPT-Cl and TBT-NCS showed similar effects on all three variants of L1210 cells. Similar measurements were also obtained when VCR, a prototypical P-gp substrate [23], was added together with the respective triorganotin derivatives on R and T cells (not shown).

IC_50_ values (median of lethal concentrations), which did not exceed 0.5 µM for any triorganotin derivative, were calculated by nonlinear regression according to Equation (1) and are summarized in Table 2, and measurements when VCR was applied together with organotin derivatives were also observed. These values demonstrated the specific effects of triorganotins on S, R and T cells as described above. VCR applied together with TBT-I and TPT-NCS potentiated cell death in R and T cells. In contrast, the presence of VCR together with TBT-Cl, TBT-Br, TBT-NCS and TPT-Cl in cultivation medium did not alter the death of R and T cells (Table 2).

**Figure 2:**
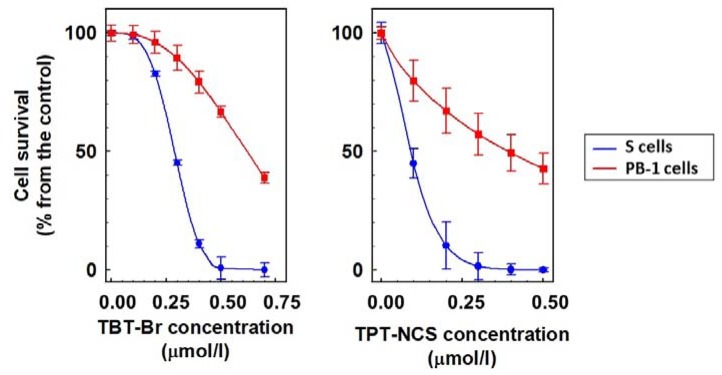
The cell death effects of TBT-Br and TPT-NCS on S and PB-1 cells. The cells’ prior measurements were 48 h cultivated in cultivation medium in the absence or presence of the respective triorganotin derivatives at different concentrations. The data were fitted by nonlinear regression according to Equation (1) using SigmaPlot 8.0 software (Systat Software, Inc., San Jose, CA, USA) and represent the means ± S.E.M. from six independent measurements. IC_50_ values of both triorganotin derivatives for induction of S cell death are summarized in Table 2. Values IC_50_ equal to 0.62 ± 0.04 μM and 0.39 ± 0.03 μM were calculated for TBT-Br and TPT-NCS induced PB-1 cell death and differ from corresponding IC_50_ values obtained for S cells on the levels *p* < 0.02.

The most pronounced differences between cell death effects on P-gp negative S cells and P-gp positive R and T cells were induced by TBT-Br (higher effectiveness on R and T than S cells) and TPT-NCS (higher effectiveness on S than R and T cells). Therefore, these two derivatives were further used for measurements of their possible selective action on neoplastic cells S as compared with normal murine pre-B cells PB-1. This experiment revealed higher cytotoxicity of both organotin derivatives on leukemia cells S that on normal cells PB-1 (Figure 2).

### 2.3. Effect of Triorganotin Derivatives on Expression and Drug Efflux Activity of P-gp

**Figure 3:**
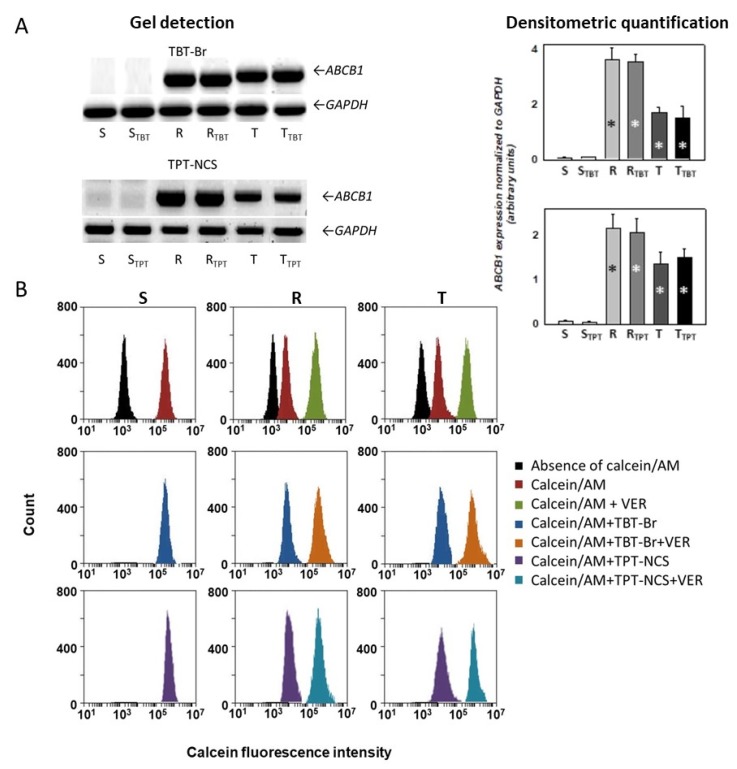
Effects of TBT-Br and TPT-NCS on expression/drug efflux activity of P-gp. (**A**) cellular levels of P-gp transcripts in S, R and T cells. The cells were cultivated in the absence and presence of either TBT-Br or TPT-NCS (both in concentration 0.05 µM). Then, the transcript levels of *abcb1* gene were estimated. Electrophoretograms (gel detection) are representative of three independent measurements. GAPDH mRNA was used as a housekeeping gene. The bands were quantified by densitometry, and the quantification is documented in column plots (densitometric quantification), in which the data are presented as the means ± S.E.M. from three independent measurements. Significance: * indicates that the values differ from the corresponding results obtained for S cells on the level *p* < 0.001; (**B**) recording of calcein retention within S, R and T cells. Calcein retention assays were performed in the absence and presence of TBT-Br, TPT-NCS (both in concentration 0.25 µM) and verapamil (Verapamil at this concentration fully inhibited the P-gp efflux activity of R and T cells [20,21,22]) (10 μM). Histograms in the same positions were obtained for S cells with the addition calcein/AM alone or together with either respective triorganotin or verapamil as well as with combination of triorganotin with verapamil. The data are representative of three independent measurements.

Both TBT-Br and TPT-NCS were further used for measurements of their ability to induce alterations in P-gp expression or drug efflux activity (Figure 3). Both P-gp positive R and T cells contain *abcb1* transcript (from mouse chromosomal gene—R, and human gene from plasmid—T). In contrast, S cells did not contain these transcripts (Figure 3A). The presence of either TBT-Br or TPT-NCS during cultivation did not induce measurable changes in the contents of P-gp gene transcripts in all three variants of L1210 cells (Figure 3A).

The drug efflux activity of P-gp was measured based on calcein retention within S, R and T cells. The principles of this assay have been described in detail elsewhere [24]. Expressive retention of calcein is visible within P-gp negative cells (Figure 3B). In contrast, the retention of this indicator is much less pronounced in P-gp positive R and T cells, in which verapamil (known P-gp inhibitor) fulfills similar calcein trapping, as is characteristic for S cells. Verapamil did not induce any changes in calcein retention within S cells. Neither TBT-Br nor TPT-NCS altered the retention of calcein within S, R and T cells (Figure 3B). Taken together, these facts indicated that neither TBT-Br nor TPT-NCS altered P-glycoprotein expression/drug efflux activity in P-gp positive R and T variants of L1210 cells. Similar results were obtained when other triorganotin derivatives were used in this type of experiment (data not shown).

### 2.4. Mechanism of Triorganotin Cell Death Effects

**Figure 4:**
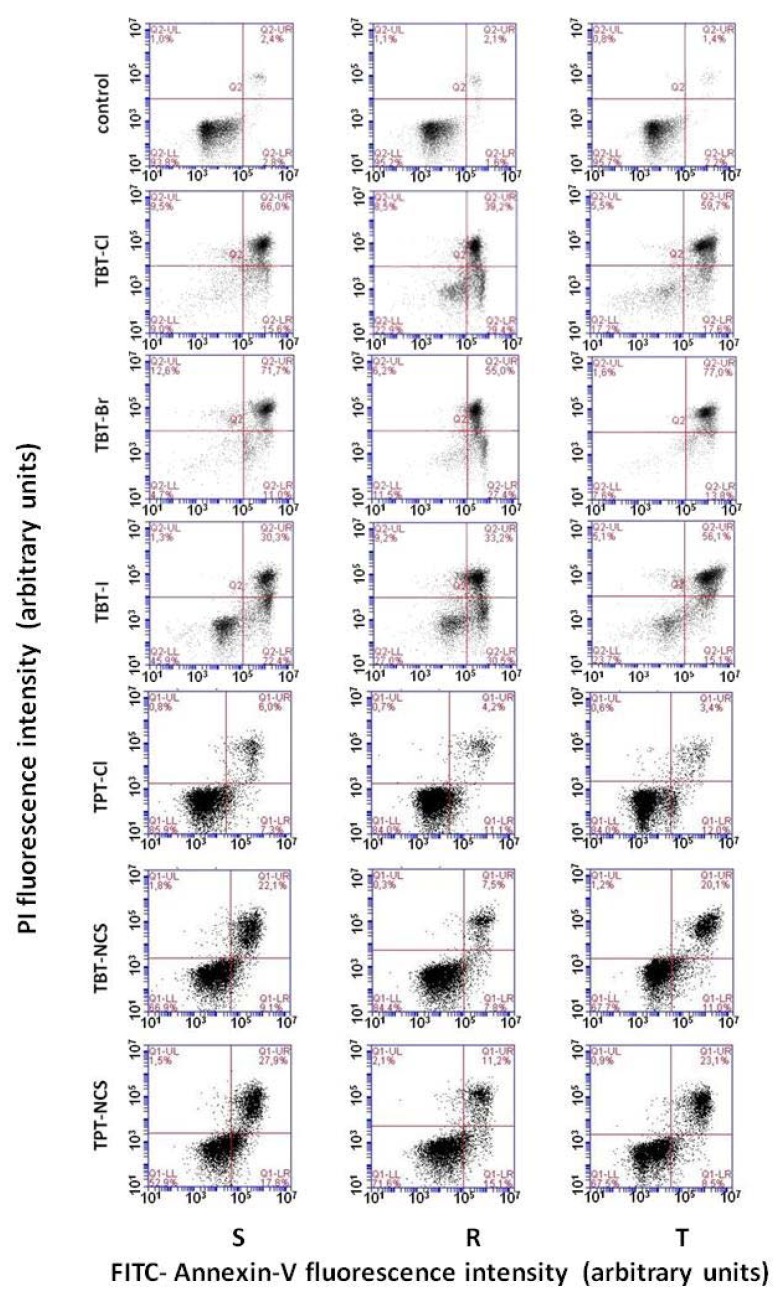
Measurement of S, R and T cells proportions in apoptosis and necrosis using a AV/PI apoptosis necrosis kit. The cells were cultivated for 24 h in cultivation medium in the absence or presence of triorganotin derivatives (0.2 µM) prior to this measurement. The dot blots are representative of three independent measurements.

Fluorescein isothiocyanate-labeled annexin V (AV) and propidium iodide (PI) were used as markers of apoptosis and necrosis, respectively. Proportions of viable S, R and T cells (i.e., cells that did not bind AV or PI) after a 24-h incubation period in cultivation medium with the absence of triorganotin derivatives (at standard cultivation condition) consistently exceeded 90% (Figure 4). The presence of triorganotin derivatives (at concentration 0.2 µM) in the cultivation medium induced an increase of either the proportion of cells labeled with AV (apoptotic cells) or the proportion of cells labeled by both AV and PI (cells in late apoptosis). The labeling of cells by PI alone (i.e., cells under necrosis) was not as pronounced (Figure 4).

**Figure 5:**
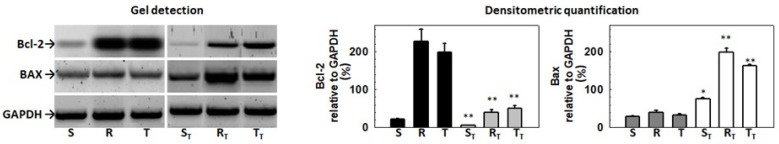
Detection of Bcl-2 and BAX protein levels in S, R and T cells incubated for 24 h in cultivation medium in the absence (S, R, and T) or presence of TBT-Br (S_T_, R_T_ and T_T_). The blots records are representative of three independent measurements. GAPDH was used as a housekeeping protein. Immunostained protein bands were quantified by densitometry, and protein quantity is documented in column graph. Data represent the mean ± S.E.M. for three independent measurements. Significance: * values differ from untreated control on the level *p* < 0.05; ** values differ from untreated control on the level *p* < 0.02.

To determine whether cell death is induced by the presence of triorganotin derivatives in the cultivation medium and is controlled by Bcl-2-mediated apoptosis, we estimated the cell content of proapoptotic and antiapoptotic members of this protein family. For this reason, we estimated the levels of Bcl-2 and BAX proteins in cells cultivated for 24 h in the absence or presence of 0.2 µM TBT-Br (Figure 5). R and T cells contain higher amounts of antiapoptotic Bcl-2 protein than S cells. Differences in the cellular content of Bax protein between P-gp negative S and P-gp positive R and T cells were not observed. The presence of TBT-Br in cultivation medium induced either a decrease of Bcl-2 proteins or an increase of BAX protein in all three variants of L1210 cells.

### 2.5. Relations between the Effectiveness of the Cell Death Effects and Lipophilicity of Triorganotins

Any direct proportionality between triorganotin cell death effects, parameterized by IC_50_ values (Table 2), and the cell lipophilicity of triorganotin derivatives, expressed as log *p* values (Table 1), was not observed, independent of whether VCR as a prototypical P-gp substrate was added together with the respective triorganotin. Linear regression revealed insignificant results for such direct relations (data not shown). When the IC_50_ values obtained for S cells were correlated with the corresponding IC_50_ values for either R or T cells, the probability levels did not fulfill the criteria for significance (Table 3). However, when the IC_50_ values for R and T cells were intercorrelated, statistically significant linear dependence was obtained (Table 3).

**Figure 6:**
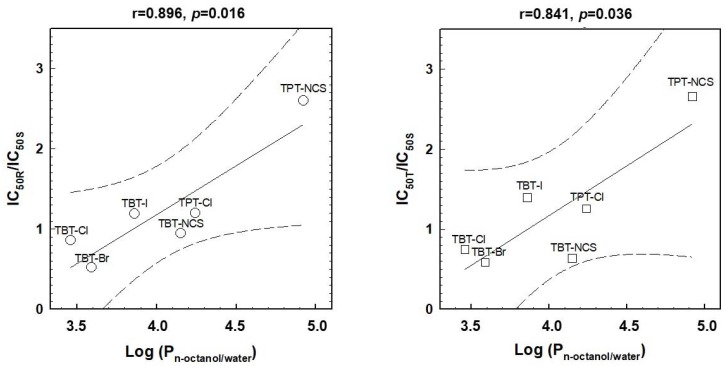
The correlations between the ratios of the IC_50_ values (from Table 1) obtained for either R versus S cells (IC_50R_/IC_50S_) or T versus S cells (IC_50T_/IC_50S_) and log P are documented in Table 1. Linear regressions were calculated using SigmaPlot graphing software (version 8.00, Systat Software GmbH, Erkrath, Germany). The *p*—two-tailed probability were calculated for the corresponding r—correlation coefficient and a sample size of 6 using the online *p*-value Calculator for Correlation Coefficients similarly to that described in Table 3. Lines: — regression line; - - - - confidence interval (level 0.99).

As described in Section 2.2, TBT-Cl or TBT-Br more effectively affected R and T cells than S cells, and TBT-I or TPT-NCS exerted the opposite behavior. This altered effectiveness could be associated with changes in the lipophilicity of triorganotins. Therefore, we correlated the ratios of the IC_50_ values for either R versus S cells (IC_50R_/IC_50S_) or T versus S cells (IC_50T_/IC_50S_) with the log *p* values for the triorganotin derivatives documented in (Table 1). The results revealed significant linear regression with r = 0.896, *p* < 0.02 for the correlation IC_50R_/IC_50S_ versus log P and r = 0.841, *p* < 0.05 for correlation of IC_50T_/IC_50S_ versus log P (Figure 6).

## 3. Discussion

Multidrug resistance represents a major obstacle in the effective treatment of neoplastic diseases including blood malignancies [5]. Until now, the diverse but well understood mechanisms of MDR have been identified [4], from which the efflux activity of overexpressed P-gp represents the most frequently occurring molecular cause [6,7]. Therefore, searching for substances that are effective also in cells with overexpressed P-gp represents an important assignment for medicinal chemistry. This was the main reason for which we have oriented ourselves to cell death effects induced by triorganotins in leukemia cells related to P-gp expression.

The results of the present study suggest that, at submicromolar concentrations, triorganotin derivatives induce strong cell death effects on either P-gp positive or P-gp negative cells (Figure 1). Consistently with our results, measurement of TPT-Cl and its hydroxide (TPT-OH)_n_ cytotoxic activity against many cancerous and normal cells revealed their effectiveness in submicromolar concentration [25]. Moreover, TBT-Cl and particularly TBT-Br induced a stronger cell death effect on P-gp positive R and T cells than on P-gp negative S cells (Figure 1, Table 2). Thus, these two derivatives showed improved effectiveness in cells with developed P-gp-mediated MDR. In contrast to TBT-Cl and TBT-Br, TBT-I and TPT-NCS showed the opposite behavior and showed a more potent attack on P-gp negative S cells than on both P-gp positive variants of L1210 cells (Figure 1, Table 2). We proved higher sensitivity of leukemia S cells to TBT-Br and TPT-NCS (chosen as model triorganotins) than to normal PB-1 cells (Figure 2). PB-1 cells represents murine pre-B cell line established from the bone marrow of a CBA/C57BL mouse [26]. These cells with B differentiation characteristics were used because we previously detect the markers of B but not T differentiation in S cells by cytochemistry [27].

Until recently, relatively little was known about the ability of triorganotin derivatives to damage neoplastic cells expressing P-gp as the frequently observed molecular cause of drug resistance. However, the present results indicated that these substances may also be effective in this situation. The presence of VCR as a P-gp prototypical substrate [23] during the cultivation of R and T cells with TBT-I and TPT-NCS significantly depresses their median effective concentration (Table 2). Together with the aforementioned reduced effectiveness of these two triorganotin derivatives on P-gp positive than P-gp negative cell (Figure 1), we can presume a role for P-gp in the depression of their cell death effects toward P-gp positive cells. Previously published data based on SwissADME numerical analysis revealed a prediction that tributyltin (IV) carboxylate compounds could be substrates for P-glycoprotein efflux [28]. However, the P-gp efflux of triorganotin derivatives could not be massive because these substances did not effectively alter the lack of calcein retention within P-gp positive R and T cells as shown for TBT-Br and TPT-NCS in Figure 3. The lack of calcein retention within P-gp expressing cells is often used as a P-gp efflux activity measure [29,30]. Therefore, triorganotin derivatives did not exert the strong competition for P-gp transport activity that is typical for potent P-gp substrate/inhibitor verapamil [31], as shown on Figure 3. Tributyltin administered via the placenta and lactation decreased renal ABCB1b expression in offspring [17], and prolonged application of this substance oppositely elevated P-gp expression/drug efflux activity in Caco-2 cells [18]. In contrast, in the present study, triorganotin derivatives did not induce any visible changes in P-gp expression in R and T cells, as documented for TBT-Br and TPT-NCS in Figure 3.

We further examined the mode of cell death induced by triorganotin derivatives. The mode of cell death induced by triorganotin derivatives was estimated using AV/PI detection, enabling the cytometric quantification of apoptotic and necrotic cells [32]. This procedure revealed the elevation of cells labeled with either AV alone or both AV and PI and only a negligible proportion of cells labeled with PI alone (Figure 4). This behavior is consistent with apoptotic cell death. Both P-gp positive cell variants (R and T) cultivated in the absence of triorganotin derivatives contained higher levels of antiapoptotic Bcl-2 protein than P-gp negative S cells (Figure 5). Coexpression of Bcl-2 and P-gp confers resistance against induction of apoptosis in leukemia cells originated from alteration in lymphoid pathway of hematopoiesis [33,34]. The initiation and progression of the intrinsic (mitochondrial) pathway of apoptosis is functionally related to the depression of antiapoptotic and elevation of proapoptotic proteins of the Bcl-2 family [35]. Cell death effects of TBT-Br in S, R and T cells are associated with a decrease in antiapoptotic Bcl-2 cell content and the elevation of the proapoptotic Bax cell content (Figure 5) in all three variants of L1210 cells. Consistent with a previous paper [9], we concluded that in mice leukemia cells, triorganotin derivatives induced apoptosis related to the Bcl-2 protein family regulatory pathway.

During the cultivation of S, R and T cells in medium containing triorganotin derivatives, these substances exist in uncharged lipophilic complexes due to pH values exceeding 7.0 [15]. The lipophilicity of these complexes (defined with calculated log *p* value in n-octanol: water, Table 1) is responsible for the ability of these substances to migrate through the cell plasma membrane and induce remarkable cell death effects in the cell internal space. However, the correlation between log P and IC_50_ values obtained for the cell death effects of triorganotin derivatives on S, R and T cells did not fulfill criteria for significance. P-gp efflux activity in R and T cells could at least partially remove the substances from the internal cell space that may operate against the cell death effects of P-gp substrates (reviewed in [7]). Moreover, P-gp induced additional resistance as a regulatory protein with antiapoptotic activity towards substances that are not suitable for P-gp efflux (reviewed in [6]). This antiapoptotic activity seems to be independent on P-gp efflux activity because it is also present in cells expressing the P-gp-efflux defective mutant [36]. Both activities of P-gp may alter R and T cell sensitivities to triorganotin derivatives. This idea is consistent with results showing that the triorganotin IC_50_ values obtained for R and T cells were significantly correlated (Table 3), indicating similarity in the induction of cell death in both P-gp positive cells by triorganotin derivatives. Theoretically, the regression line should diagonally pass through the origin (i.e., with 45° angle with abscissa). The intercept of the regression line on the ordinate is negligibly low and the slope is 0.928 (Table 3), indicating a 42.9° angle with abscissa; thus, this regression line is close to the theoretical course. The IC_50_ values obtained for the cell death effects of triorganotins on S cells that do not contain P-gp did not correlate with the corresponding data obtained for R and T cells.

A good example of P-gp influence on cell death effect is that TBT-I and TPT-NCS induced less efficient cell death on R and T than on S cells, and this feature could be reversed by P-gp prototypical substrate VCR (Table 2). Alterations in the effectiveness of the respective triorganotin derivatives on P-gp positive cells (R and T) and P-gp negative S cells could be expressed by IC_50R_/IC_50S_ ratios to compare R and S or IC_50T_/IC_50S_ to similarly compare T and S, as previously used in a QSAR study of P-gp substrates [37]. Both ratios were significantly correlated with the log *p* values of triorganotin derivatives (Figure 6). Therefore, it could be assumed that the presence of P-gp in L1210 cells could negatively influence the effectiveness of triorganotin derivatives, and this effect is more pronounced when derivatives with higher lipophilicity (log P) are applied.

## 4. Materials and Methods

### 4.1. Chemicals

Tributyltins (TBT-Cl, TBT-Br and TBT-I) and triphenyltin chloride (TPT-Cl), VCR, verapamil, calcein/AM and [3-(4,5-dimethylthiazol-2-yl)-2,5-diphenyltetrazolium bromide] (MTT), were purchased from Sigma-Aldrich (San Diego, CA, USA). An apoptosis necrosis kit based on cell staining with annexin V linked to fluorescein isothiocyanate and propidium iodide and a proteome extraction kit were obtained from Calbiochem (Sigma-Aldrich, San Diego, CA, USA). Rabbit polyclonal antibodies against Bcl-2, BAX, GAPDH (as an internal standard) and goat anti-rabbit antibody linked with horseradish peroxidase were from Santa Cruz Biotechnology (Dallas, TX, USA). The ECL detection system was obtained from GE Healthcare Europe GmbH (Vienna, Austria). Components of the cultivation media RPMI 1640 media, L-glutamine, fetal bovine serum and gentamycin were purchased from Gibco (Langley, OK, USA). All other commercially available chemicals were of analytical grade and were obtained from Sigma-Aldrich.

Tributyltin isothiocyanate (TBT-NCS) and triphenyltin isothiocyanate (TPT-NCS) were prepared by refluxing alcohol solutions of the respective tin chlorides with KSCN used in excess (1.5 equiv.), as previously described [38,39]. The obtained crude products were purified either by fractional bulb-to-bulb distillation (TBT-NCS: b.p. 160 °C, 0.25 mm [38]) or recrystallization from CH_2_Cl_2_–n-hexane (TPT-NCS: m.p. 165–167 °C [39]). The structures of the prepared compounds were confirmed by IR and NMR techniques. IR spectra were recorded on a SmartMIRacle ATR Zn/Se for Nicolet Impact 410 FT-IR (Thermo Scientific, Langenselbold, Germany). NMR spectra were measured on a JEOL ECZR-400 MHz spectrometer (JEOL, Ltd., Akishima, Tokyo, Japan). Experiments were carried out at 25 °C, chemical shifts are reported in δ parts per million (ppm) and J values in Hz. The residual solvent signals of CDCl_3_, were used as a reference.

TBT-NCS spectral characteristics: ^1^H NMR (CDCl_3_, 400 MHz) δ/ppm: 1.69–1.59 (m, 2H), 1.35 (sext, ^3^*J* = 7.3 Hz, 2H), 1.31–1.25 (m, 2H), 0.93 (t, ^3^*J* = 7.3 Hz, 3H); ^13^C NMR (CDCl_3_, 101 MHz) δ/ppm: 142.9, 27.7, 26.8, 16.3, 13.5; ^117^Sn NMR (CDCl_3_, 142 MHz) δ/ppm: 84.41; IR (neat) ṽ/cm^−1^: 2954 *m*, 2919 *m*, 2870 *m*, 2858 *m*, 2067 *s*, 1456 *w*, 1416 *w*, 1377 *w*, 1341 *w*, 1292 *w*, 1180 *w*, 1076 *w*, 1024 *w*, 960 *w*, 867 *w*, 668 *w*.

TPT-NCS spectral characteristic: ^1^H NMR (CDCl_3_, 400 MHz) δ/ppm: 7.69–7.63 (m, 6H), 7.56–7.48 (m, 9H); ^13^C NMR (CDCl_3_, 101 MHz) δ/ppm: 145.6, 136.2, 135.3, 131.0, 129.4; ^117^Sn NMR (CDCl_3_, 142 MHz) δ/ppm: −100.00; IR (neat) ṽ/cm^−1^: 3044 *w*, 2090 *s*, 2039 *m*, 1931 *w*, 1480 *m*, 1429 *m*, 1374 *w*, 1332 *w*, 1301 *w*, 1261 *w*, 1190 *w*, 1158 *w*, 1073 *w*, 1063 *w*, 1021 *w*, 997 *m*, 724 *m*, 691 *m*.

The purities of the prepared triorganotin compounds (tributyltin isothiocyanate and triphenyltin isothiocyanate) were better than 97%, as determined by NMR.

### 4.2. Cell Culture Conditions

The following three L1210 cell variants were used in the present study: (i) S-drug-sensitive parental cells were obtained from the Leibniz-Institut DSMZ-Deutsche Sammlung von Mikroorganismen und Zellkulturen GmbH (Braunschweig, Germany) ACC-123; (ii) R—P-gp-positive drug-resistant cells overexpressing P-gp after selection with VCR [20]; and (iii) T—P-gp-positive drug-resistant cells overexpressing P-gp following stable transfection with the P-gp gene [21], using the Addgene plasmid 10,957 (pH aMDRwt), a retrovirus encoding the full-length P-gp cDNA [40]. Murine pre-B cells PB-1 established from the bone marrow of a CBA/C57BL mouse obtained from the Leibniz-Institut DSMZ-Deutsche Sammlung von Mikroorganismen und Zellkulturen GmbH (ACC-241) were used as normal cells without features consistent to leukemia transformation. The cells i. S, R and T (inoculums 1 × 10^6^ cells) were cultured in 4 cm^3^ RPMI 1640 media with l-glutamine (1 mg cm^−3^), containing 4% fetal bovine serum and 1 μg cm^−3^ gentamycin, in a humidified atmosphere with 5% CO_2_ and air at 37 °C for 48 h; ii. PB-1 (inoculums 1 × 10^6^ cells) were cultured in 4 mL of 85% McCoy’s 5A medium supplemented with 15% of non-heat inactivated fetal bovine serum, 50 ng/mL human IL-7, 0.4× MEM vitamins, 1× MEM non-essential amino acids, 0.5× essential amino acids, 1 mM sodium pyruvate, 0.12% sodium bicarbonate, 2 mM l-glutamine and 50 μM mercaptoethanol. All components of this medium were purchased by Sigma-Aldrich. PB-1 cells were passaged in a humidified atmosphere with 5% CO_2_ and air at 37 °C for 48 h. All cells (S, R, T and PB-1) were cultivated in the absence or presence of triorganotin derivatives (TBT-Cl, TBT-Br, TBT-I, TBT-NCS, TPT-Cl and TPT-NCS) at a concentration range of 0.05–0.5 µM. The numbers of viable cells after each passage were counted using a CASY Model TT Cell Counter (Roche Applied Sciences, Madison, WI, USA). R cells were cultured for two passages without VCR prior to the experiments.

### 4.3. Cell Death Effects of Triorganotin Derivatives on S, R, T and PB-1 Cells

The cells (5 × 10^4^ cells/well) were cultured in the presence or absence of the respective triorganotin derivative with or without VCR (1.2 µM) in 96-well cell culture plates. Triorganotin derivatives and VCR were directly added to 200 μL of culture media. After 24 and 48 h, the cell viability was assessed using the MTT assay [41], which was performed by adding MTT ([3-(4,5-dimethyldiazol-2-yl)-2,5-diphenyltetrazolium bromide]) to a final concentration of 0.25 mg cm^−3^ per well. The cells were then incubated with MTT for 2 h. Next, the plates were centrifuged for 15 min (2500 rpm, 5000× *g*), and the cell sediment was extracted with dimethyl sulfoxide. The absorbance at 540 nm was measured using a Universal Microplate Spectrophotometer mQuant (BioTek Instruments, Inc., Winooski, VT, USA). Dose-response curves were fitted according to an exponential decay equation (Equation (1)) by non-linear regression, as previously described [22]:

N = 100% × exp[ln(0.5) × (c/IC_50_)] (1)

where N represents the percentage (from a control in the absence of triorganotin derivative) of viable cell after culturing in the presence of respective triorganotin derivative at a concentration c. The IC_50_ is the concentration of a substance when N = 50%. The experimental data were fitted by nonlinear regression using the SigmaPlot graphing software (version 8.00, Systat Software GmbH, Erkrath, Germany). Statistical significance was analyzed using an unpaired Student’s *t*-test.

### 4.4. Reverse Transcription Polymerase Chain Reaction (RT-PCR) Detection of P-gp Expression

The S, R and T cells were incubated in the absence and presence of either TBT-Br or TPT-NCS (both in concentration 0.05 µM), and then, total RNA was isolated using Trizol Reagent (Life Technology, Bratislava, Slovakia) according to the manufacturer’s instructions. Reverse transcription was performed with 2 µg of DNase I-treated RNA and the RevertAid^TM^ H Minus First-Strand cDNA Synthesis Kit (Thermo Scientific, Langenselbold, Germany) according to the manufacturer’s protocol. PCR was performed in a total volume of 25 µL using a PCR kit according to the manufacturer’s protocol (Thermo Scientific). The expression of *GAPDH* was used as an internal standard. After treating the samples at 94 °C for 3 min to inactivate the reverse transcriptase, the samples were subjected to 30 cycles of 95 °C for 30 s, followed by 57 °C for 30 and ending with 72 °C for 90 s, and a final extension of 72 °C for 10 min. The PCR products were separated on a 1.5% agarose gel (Invitrogen, Life Technology, Bratislava, Slovakia) and visualized using GelRed^TM^ nucleic acid gel stain (Thermo Scientific). The gels were stained, and the bands were quantified using the Typhoon 9210 imaging system (GE Healthcare). The data were expressed as the relative level of each mRNA normalized to that of GAPDH. Statistical significance was analyzed using unpaired Student’s *t*-test. The following primer sequences were used in the present study: *GAPDH*: 5′-AAC TTT GTC AAG CTC ATT TCC-3′ and 5′-GCA GCG AAC TTT ATT GAT GGT-3′, which produced a 267 bp product; human *MDR1*’: 5′-AAG TTG TAT ATG GTG GTG GGA ACT-3′ and 5′-AAT TTT GTC ACC AAT TCC TTC ATT-3′, which produced a 429-bp product and were used for T cells; and mouse *MDR1:* 5′-AGG TAG AGA CAC GTG AGG TCG-3 and 5′-CAG CCA ACC TGC ATA ACG-3′, which produced a 453-bp product and were used for R cells.

### 4.5. Estimation of P-gp Transport Activity by Calcein/AM Retention Assay

P-gp transport activity was measured using a calcein retention assay [42,43]. The cells were spun down (500× *g*) and washed two times in phosphate-buffered saline (PBS, Sigma-Aldrich) containing 0.2% BSA to remove the drugs. The cells were then resuspended in 0.5 cm^3^ of the same solution. Calcein/AM (at final concentration: 0.1 μM) was added directly to the solution with the cells. Calcein retention assays were performed in the absence or presence of TBT-Br, TPT-NCS (both in concentration 0.25 µM), and verapamil (10 μM) added directly to the solution with the cells. The samples were subsequently incubated for 20 min at 37 °C in a CO_2_ incubator. After incubation, propidium iodide (Sigma-Aldrich) was added at a final concentration of 0.9 μM, and the cells were incubated for an additional 10 min. The cells were then washed twice with ice-cold PBS. Fluorescence was measured using an Accuri C6 flow cytometer (BD Bioscience, San Jose, CA, USA). Only viable, non-propidium iodide-stained cells (more than 92% in each case) were counted.

### 4.6. Measurement of S, R and T Cells’ Proportions in Apoptosis and Necrosis Using

The cells (S, R and T) were cultivated for 24 h in cultivation medium in the absence or presence of triorganotin derivatives (0.2 µM) prior to this measurement. After passaging, the cells were harvested and used for estimation by apoptosis and necrosis using a fluorescein isothiocyanate-annexin V and propidium iodide kit. According to the procedure described by the manufacturer, the cells were washed twice with PBS and gently resuspended in binding buffer (obtained from the manufacturer) containing 0.5 μg cm^−3^ fluorescein isothiocyanate-annexin V. The mixture was then incubated in the dark at room temperature for 15 min, followed by centrifugation (2500 rpm, 15 min). The resulting sediments were re-suspended in binding buffer and propidium iodide (final concentration 0.6 μg cm^−3^) and added to each sample. The samples were analyzed by flow cytometry using an Accuri C6 flow cytometer (BD Bioscience, San Jose, CA, USA). In this assay, the viability of the cells was evaluated as follows [22]: (i) cells not stained by both fluorescein isothiocyanate-annexin V and propidium iodide represented viable cells; (ii) cells stained by fluorescein isothiocyanate-annexin V or propidium iodide were considered apoptotic or necrotic cells, respectively; and (iii) cells stained by both fluorescein isothiocyanate-annexin V and propidium iodide were fully damaged death cells in the late phases of apoptosis or necrosis.

### 4.7. Western Blot Procedures

The proteins (Bcl-2, BAX and GAPDH) were detected by blotting using a specific primary antibody in whole cell lysate isolated from S, R and T cells incubated for 24 h in the absence or presence of either TBT-Br (0.2 µM). Total cell proteins were obtained using the Extraction Kit according to the manufacturer’s protocol. The proteins from (30 μg per line) were separated via sodium dodecyl sulfate polyacrylamide electrophoresis (SDS-PAGE) on 12% polyacrylamide gradient gels using the protocol published by Laemmli [44]. The proteins were subsequently transferred by electroblotting onto nitrocellulose membranes (GE Healthcare Europe GmbH, Vienna, Austria) using the protocol published by Towbin et al. [45]. The polyclonal antibodies against Bcl-2, BAX and GAPDH (all in dilution 1:200) and goat anti-rabbit antibody conjugated with horseradish peroxidase (in dilution 1:500) were used as primary and secondary antibodies, respectively.

## 5. Conclusions

P-gp-positive variants of L1210 cells (R and T) are more sensitive to TBT-Cl and TBT-Br than P-gp negative S cells. In contrast, TBT-I and TPT-NCS induce a more pronounced cell death effect on P-gp negative S cells than on both P-gp positive (R and T) variants of L1210 cells. Triorganotin derivatives induced a cell death effect on all variants of L1210 cells applied in the present study at submicromolar levels. The mode of cell death seems to be apoptosis related to the Bcl-2 protein regulatory pathway. All triorganotins applied in the present study fail to alter either P-gp expression measured by RT-PCR or P-gp efflux activity measured by the retention of calcein within the cells. We concluded that triorganotins represent efficient substances suitable to alter the viability of malignant cells in cases where P-gp-mediated drug resistance is developed.

## Figures and Tables

**Table 1:** Structures and lipophilicity of triorganotin derivatives.

Compound	Abbreviation	Structure ^1^	Log P ^2^
Tributyltin chloride (*Chlorotributyltin, Chlorotributylstannane*)	TBT-Cl	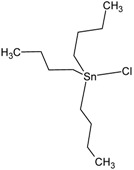	3.46
Tributyltin bromide (*Bromotributyltin, Bromotributylstannane*)	TBT-Br	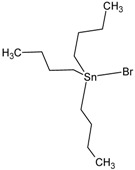	3.59
Tributyltin iodide (*Iodotributyltin, Iodotributylstannane*)	TBT-I	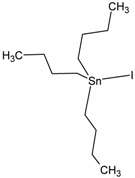	3.86
Tributyltin isothiocyanate (*Isothiocyanatotributyltin, Isothiocyanatotributylstannane*)	TBT-NCS	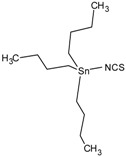	4.15
Triphenyltin chloride (*Chlorotriphenyltin, Chlorotriphenylstannane*)	TPT-Cl	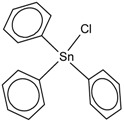	4.24
Triphenyltin isothiocyanate (*Isothiocyanatotriphenyltin, Isothiocyanatotriphenylstannane*)	TPT-NCS	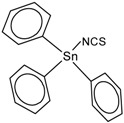	4.92

^1^ The 3D structure of the molecules was calculated using ACD/ChemSketch (Freeware) software http://www.acdlabs.com/). ^2^ Calculated using the Molinspiration online log P calculator. According to information in this web (http://www.molinspiration.com/cgi-bin/properties), the values for log P measured were compared with those for log P predicted for 12,202 molecules for which error of predicted log P did not exceed 0.25 for 50.5%; 0.5 for 80.2% and 1.0 for 96.5%

**Table 2:** The IC_50_ values for the growth inhibition of S, R and T cells by triorganotin derivatives.

IC50 for	S	R	R_v_	T	T_v_
Substances			(in μM)		
TBT-Cl	0.29 ± 0.03	0.25 ± 0.02 *	0.23 ± 0.05 *	0.21 ± 0.05 **	0.21 ± 0.04 **
TBT-Br	0.29 ± 0.05	0.15 ± 0.05 **	0.15 ± 0.04 **	0.17 ± 0.07 **	0.16 ± 0.04 **
TBT-I	0.14 ± 0.02	0.18 ± 0.03 *	0.15 ± 0.02 ^+^	0.21 ± 0.04 **	0.16 ± 0.03 ^+^
TPT-Cl	0.37 ± 0.09	0.45 ± 0.09	0.44 ± 0.10	0.47 ± 0.11	0.48 ± 0.12
TBT-NCS	0.31 ± 0.09	0.30 ± 0.05	0.33 ± 0.07	0.20 ± 0.05 *	0.20 ± 0. 04 *
TPT-NCS	0.09 ± 0.02	0.24 ± 0.04 **	0.10 ± 0.02 ^++^	0.24 ± 0.06 **	0.09 ± 0.03 ^++^

The IC_50_ values were calculated by nonlinear expression of experimental data according to Equation (1) and are expressed as computed values ± S_d_. for 6 independent mesaurements. Significance: ** and * indicate that the data differ from the corresponding results obtained for S cells on the levels *p* < 0.02 and *p* < 0.05, respectively. ^++^ and ^+^ indicate that the data differ from corresponding results obtained in the absence of VCR on the levels *p* < 0.02 and *p* < 0.05, respectively. S, R and T indicate the variants of L1210 cells, R_v_ and T_v_ indicate R and T cells cultivated with the respective triorganotin derivatives in the presence of VCR (1.2 µM) that fully blocked the proliferation of S cells and did not considerably affect the proliferation of R and T cells.

**Table 3:** Linear regression of the IC_50_ values for triorganotin cell death effects on S, R and T cells.

Correlation	B_0_	B_1_	r	*p*	Significance
S vs. R	-	-	0.582	0.226	Insignificant
S vs. T	-	-	0.425	0.401	Insignificant
R vs. T	0.008	0.928 (42,9°)	0.902	0.014	significant (*p* < 0.02)

Symbols: B_0_, B_1_ represents Y intercept and slope of regression line, respectively, calculated using SigmaPlot graphing software (version 8.00, Systat Software GmbH, Erkrath, Germany); the values in parentheses represent the angle of regression line with abscissa, r correlation coefficient; *p* two-tailed probability calculated for the corresponding r and sample size 6 using online *p*-value Calculator for Correlation Coefficients: (http://www.danielsoper.com/statcalc/calculator.aspx?id=44); S, R and T indicate that the IC_50_ values of the respective triorganotins obtained for S, R and T cells were used for correlation.
